# Uncovering the Floral Origins of Honey Bee Pollen in Colombian Tropical Dry Forest: A Low-Cost DNA Barcoding Approach Reveals *Cactaceae* Dominance

**DOI:** 10.3390/plants14233652

**Published:** 2025-11-30

**Authors:** Maryuri Lobo-Torres, Diana C. Mantilla-Escalante, Brayan J. Anaya, Diego F. Tirado, Claudia M. Arenas-Gómez

**Affiliations:** 1 Dirección Académica, Universidad Nacional de Colombia, Sede de La Paz, La Paz 202017, Colombia; mlobot@unal.edu.co (M.L.-T.); or branaya@ucm.es (B.J.A.); 2 Grupo de investigación GIND, Seccional Cartagena, Universidad del Sinú Elías Bechara Zainúm, Cartagena de Indias 130001, Colombia; diana.mantilla0819@gmail.com; 3 Department of Pharmaceutics and Food Science, School of Pharmacy, Complutense University of Madrid, 28040 Madrid, Spain

**Keywords:** apiculture, *Apis mellifera*, flora, gene *matK*, gene *rbcL*, pollination

## Abstract

Characterizing the botanical composition of pollen is essential to understanding the floral resources used by bees. While microscopy is the traditional method, it is time-consuming and limited in taxonomic resolution. Molecular tools such as DNA barcoding offer a more precise and cost-effective alternative for identifying plant taxa in mixed pollen samples. This study implemented a preliminary and cost-effective molecular approach to identify the botanical origin of pollen stored in bee bread from *Apis mellifera* hives in a tropical dry forest fragment in La Paz, Cesar, using *rbcL* and *matK* genes as markers. The chloroplast markers *rbcL* and *matK* were amplified and Sanger-sequenced from three independent bee hives, each processed in duplicate as technical replicates. The BLAST+ 2.17.0 results from Sanger sequences showed a sequence identity ranging from 89%–99%, with *rbcL* showing higher and more consistent matches than *matK*, suggesting stronger discriminatory power, while the lower identity in one hive indicated a more complex pollen mixture. However, *matK* detected a greater number of taxa overall (*i.e.*, 70% of the total, 64 genera) compared with *rbcL* (*i.e.*, 50%, 46 genera). Both markers overlapped in approximately 20% of the taxa, most of which (*i.e*., 94%) belonged to the family Cactaceae. This indicated that, although *rbcL* provided more reliable matches, *matK* contributed to broader taxonomic coverage, highlighting the complementarity of both markers for mixed pollen analyses. This approach highlights its value as an exploratory tool prior to applying high-throughput sequencing strategies. Furthermore, such studies may support the development of local honey brands by validating that their products originate mainly from the biodiversity of tropical dry forests, an ecosystem currently at risk, thereby conferring both ecological and market value.

## 1. Introduction

Among living pollinators, bees are the main and most effective group [1], playing an important role in keeping ecosystem diversity [2,3]. This is because, while looking for nectar, bees also collect and move pollen between flowers [4], helping with cross-pollination—a key process for many flowering plants to reproduce [1]. Some of the pollen is taken back to the hive and stored as bee bread, a fermented mix of pollen, nectar, and microbes from gland secretions, which is an important food source for the colony [5]. Within this framework, studies on flora and taxonomy based on pollen morphological analysis have traditionally been conducted using microscopy techniques [6]. This method has enabled detailed observations that facilitate the identification of floral sources used by bees, such as *Apis mellifera* [7], as pollen grains exhibit specific morphological characteristics associated with their botanical origin [8]. However, microscopy presents challenges when distinguishing species with similar pollen morphology, in addition to being time-consuming and requiring specialized personnel [9]. Furthermore, it offers limited taxonomic resolution [10]. To address these limitations, the use of molecular tools such as DNA barcoding emerges as a more precise, cost-effective, and efficient technique than traditional palynology, enabling the analysis of environmental samples like mixed pollen [11,12]. This technique is based on the amplification and sequencing of conserved genetic regions, allowing for more accurate taxonomic classification [10], even in complex samples such as bee bread [9]. Despite these advances in molecular marker applications, their use in Colombian tropical dry forests remains insufficient, particularly for complex biological matrices like bee-collected pollen. Most studies in these regions have focused on floristic inventories [13,14] or classical palynology, leaving a significant gap in the molecular characterization of pollen spectra [15].

Therefore, the development and implementation of molecular methods for analyzing bee-collected pollen is necessary to characterize the flora in Colombia’s tropical dry forest regions. Consequently, this study implemented a preliminary and cost-effective molecular approach to identify the botanical origin of pollen stored in bee bread from *A. mellifera* hives in a tropical dry forest fragment in La Paz, Cesar, using *rbcL* and *matK* genes as markers. The research sought to provide reference information for stakeholders interested in biodiversity and beekeeping in Colombia, which could enable local honey producers to authenticate the botanical and geographical origins of their honey, ultimately increasing its market value.

## 2. Results

### 2.1. Disruption Method Performance

**Figure 1:**
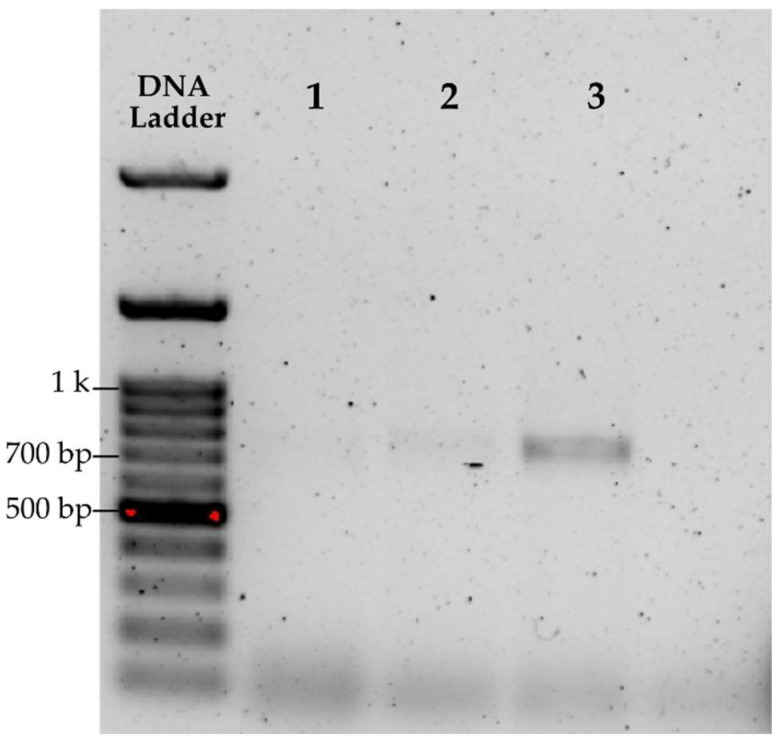
Electrophoresis of *matK* PCR products on 1% agarose gel using different disruption methods: (**1**) mortar grinding, (**2**) glass beads, (**3**) cordless homogenizer.

A preliminary, non-statistical evaluation was first conducted using only one sample to assess the efficiency of different cell lysis methods and to verify PCR amplification of the extracted DNA with the *matK* gene. The methods that showed the best performance were the cordless homogenizer for microtubes and mortar maceration (see Table 1). The most intense band corresponding to the *matK* gene of approximately 700 bp (see Figure 1, lane labeled 3) was obtained with the homogenizer, whereas samples processed with mortar and beads showed, respectively, a very faint band and a slightly more pronounced band (see Figure 1, lanes labeled as 1 and 2).

### 2.2. Quantification of DNA Yield and Purity 

The results presented in Table 2 show that bee bread samples exhibited high nucleic acid concentrations through spectrophotometry, with yields ranging from 910 ng/μL to 1387 ng/μL. These samples demonstrated A260/A280 ratios of roughly 2.1 and A260/A230 ratios of about 2, indicating DNA purity. Similarly, bee bread samples showed good yield levels and purity. In contrast, the commercial pollen sample displayed a significantly lower yield (*i.e.*, 327 ng/μL) compared to the other pollen sources analyzed.

### 2.3. PCR Amplification

**Figure 2:**
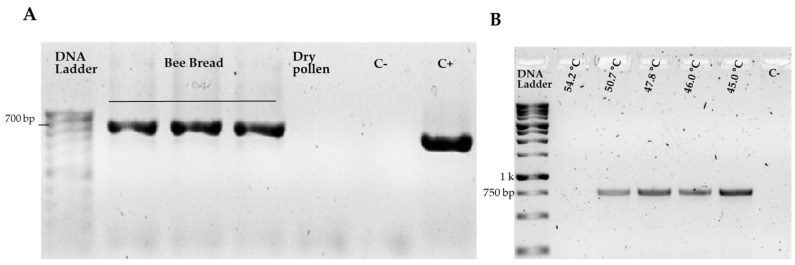
PCR result with *rcbl* and *makt* genes. Agarose gel electrophoresis (1%) showing the amplified PCR products (**A**) PCR amplification products of the *rbcL* region using bee pollen DNA, showing fragments near 700 bp. C−: negative control; C+: positive control corresponding to *Guazuma ulmifolia* (*Guácimo*) DNA; (**B**) *matK* region amplicons with annealing temperature gradient (45 °C to 60 °C), revealing fragments near 750 bp. C−: negative control.

The selected method for pollen grain disruption was validated through DNA extraction, quantification, and amplification, followed by PCR product verification via electrophoresis. Using DNA extracted from bee bread samples, the amplification of the chloroplast genes *rbcL* and *matK* was exclusively evaluated. During protocol efficiency testing with bee bread and commercial pollen samples, no *rbcL* gene amplification was detected for the latter. In contrast, the same genetic region consistently amplified under identical conditions with bee bread DNA, revealing bands near 700 bp (see Figure 2A). However, amplification of the chloroplast *matK* gene with bee pollen DNA required additional optimization due to the initial absence of amplified products when applying the same thermal conditions as for *rbcL*. The thermal profile with a hybridization temperature gradient ranging from 45 °C to 60 °C revealed optimal temperatures, showing more intense bands at 45 °C with fragments of approximately 750 bp within the expected size range (see Figure 2B). Although faint bands were detected at 50.7 °C, this hybridization temperature was employed for subsequent analyses to avoid using lower temperatures that might have generated nonspecific amplifications. Following standardization, all six samples (three biological samples, each with two technical replicates) were successfully amplified with both markers by PCR and used for Sanger sequencing.

### 2.4. Molecular Identification of Bee Bread Samples

A total of 92 genera belonging to 18 families were identified (see Supplementary File Table S1). The BLAST analysis of the 20 top matches with the sequences obtained from the three hive samples using the *matK* and *rbcL* markers showed high similarity to reference plant sequences in the NCBI database (see Table 3). For *matK*, the percent identity ranged from 89% to 97%, with E-values between 0.0 and 2.3 × 10^−114^ and total scores from 826 to 1235. The lower identity observed in Hive 2 (*i.e.*, 89%) suggested the presence of a more complex mixture of plant DNA. In contrast, the *rbcL* sequences had higher identity values (98%–99%) with E-values of 0.0 and total scores exceeding 1400, indicating stronger and more reliable matches.

The most representative results from BLAST were the identification of genera that belonged to the family Cactaceae, accounting for 61% of the total taxa. *Leuenbergeria*, *Pereskia*, and *Rhodocactus* were the most frequent genera in the main BLAST analysis results for most samples, showing the highest identity and query coverage values (see Table 4), indicating highly significant and reliable matches. It is noteworthy that some of the taxa identified (6 of 18) through BLAST comparisons correspond to plant families distributed in other regions of the world (see Table S1).

**Figure 3:**
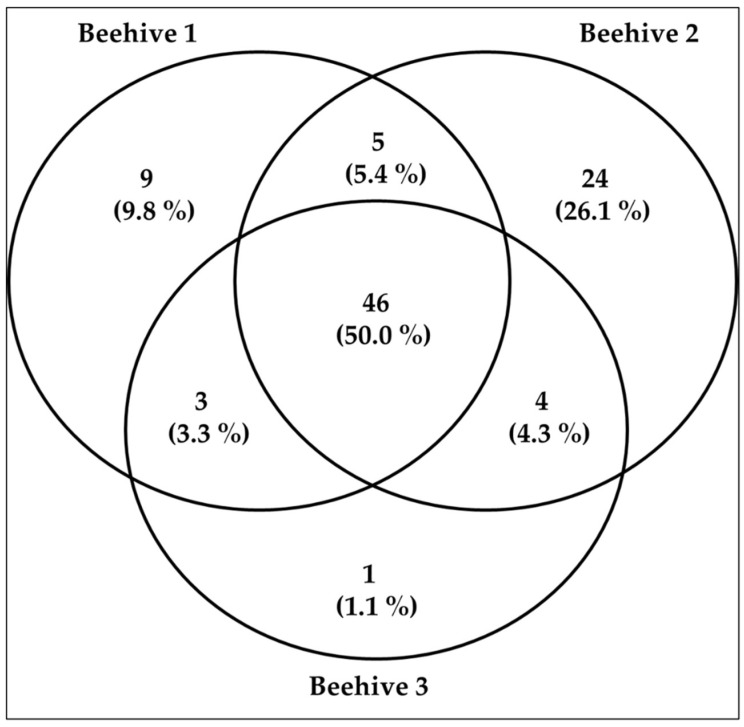
The number of plant taxa at the genus level identified by the beehive and shared among them.

The number of genera varied among hives, being the highest in Hive 2 (79 genera), followed by Hive 1 (63 genera), and Hive 3 (54 genera). Some taxa were shared across hives, while others were exclusive to a single hive (see Figure 3 and see Supplementary Materials Table S2).

**Figure 4:**
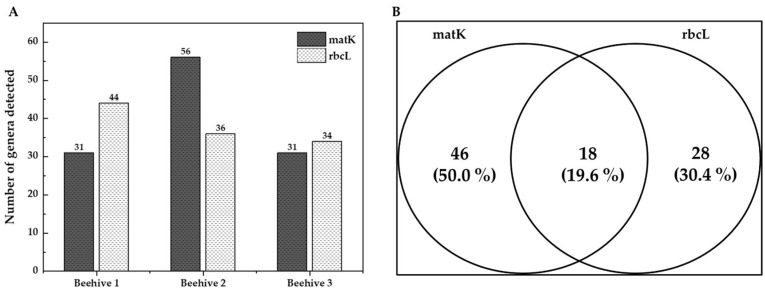
Plant taxa identified in bee bread by hive and DNA barcode marker. (**A**) Number of plant taxa identified by the beehive. (**B**) Number of exclusive and shared plant taxa at the genus level identified from bee bread samples using DNA barcoding with *matK* and *rbcL* markers, and per marker.

Regarding marker performance, *matK* identified a greater number of genera in Hive 2, whereas *rbcL* did so in Hive 1. When both markers were considered together, Hive 2 exhibited the highest number of taxa compared to the others (see Figure 4A). When evaluating the two genes independently (without distinguishing between hives), *matK* detected 70% of the total taxa identified (64 genera), while *rbcL* revealed 50% (46 genera) (see Figure 4B). Both markers coincided in approximately 20% of the taxa, 94% of which belonged to the family Cactaceae (see Supplementary Materials, Table S1).

### 2.5. Pollen Micrographs

Different palynomorphs were observed (see Supplementary Materials, Figures S1 and S2), for which a taxonomic approximation at the family level was performed, considering the characteristics of the exine ornamentation (outer layer) and visible apertures. These records were used as comparative references with respect to the molecular analysis results. Microscopic analysis enabled the identification of pollen grains from several plant families, including Fabaceae, Malvaceae, and Asteraceae. In contrast, molecular analysis based on BLAST results revealed additional genera belonging to families that were not detected microscopically. Among the 92 genera identified through molecular analysis, some likely correspond to the families observed microscopically; for instance, 3.26% (n = 3) of the genera belonged to Fabaceae, 2.17% (n = 2) to Malvaceae, 1.09% (n = 1) to Asteraceae, and 60.87% (n = 56) to Cactaceae.

## 3. Discussion

Molecular identification of bee bread as a source of pollen collected by bees represents a key tool for estimating the floristic diversity of an area [16,17]. Although the yield values showed variability, this may be associated with differential floral composition, the degree of pellet compaction, or the presence of inhibitory compounds [18,19]. Despite this, pollen collected by bees has been widely used in molecular identification studies such as DNA barcoding [8,10,20]. However, DNA release from pollen may be affected by its highly resistant outer layer; therefore, selecting an effective mechanical disruption method—such as a microtube homogenizer—is an important step for studies with pollen samples [21,22].

The BLAST results and marker performance revealed consistent differences between *matK* and *rbcL*, as well as between hives. Figure 3 clearly shows that at least 50% of the genera were shared between the hives. However, some degree of heterogeneity is also evident, as shown in the Figure 3: Hive 1 contained 9 unique genera, Hive 2 exhibited 24 unique genera, and Hive 3 displayed a single exclusive genus. This variation in hive-specific genera could suggest differences in foraging patterns or local resource availability. It is possible that the high discriminatory power of the *rbcL* gene allows the detection of genera that appear exclusively in each hive, reinforcing the need to use two complementary markers.

The *rbcL* gene showed higher sequence identity values (98%–99%) and total scores (>1400), indicating greater discriminatory reliability. In contrast, *matK* yielded slightly lower identity values (89%–97%), particularly in Hive 2, suggesting the presence of a more complex mixture of plant DNA and supporting the discriminatory effectiveness of these loci could vary across plant groups, as some markers perform better for specific taxonomic lineages.

**Figure 5:**
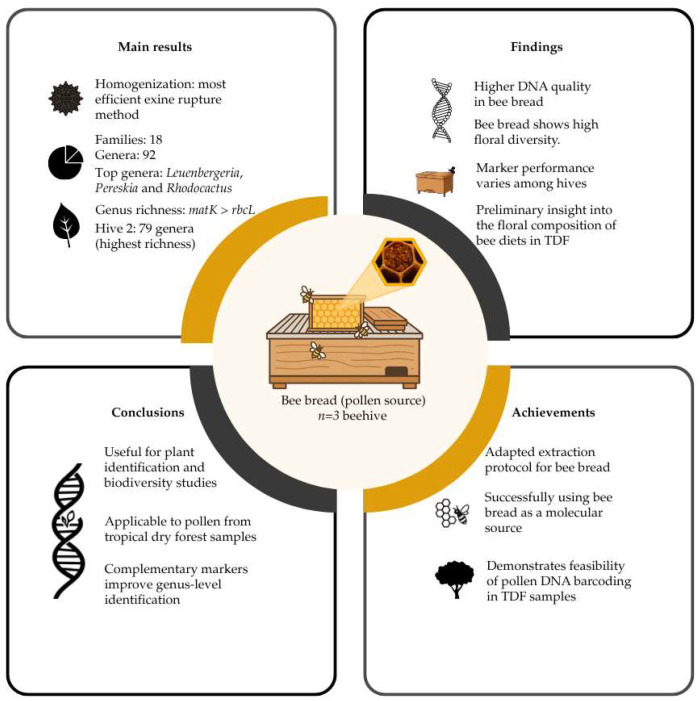
Schematic summary of the main results, findings, conclusions, and achievements of this study. TDF: Tropical Dry Forest.

Nevertheless, *matK* identified a greater number of genera overall compared to *rbcL*. This pattern suggests that, although *rbcL* provides more consistent and accurate matches, *matK* offers broader taxonomic coverage, which is consistent with previous studies highlighting *matK*‘s high genetic variation and discriminatory power for differentiating angiosperms [23,24,25,26]. These findings highlight the complementary nature of the two markers and support their combined use for more comprehensive molecular characterization of mixed pollen samples, as has been reported [25,27] (see Figure 5). This approach reduces identification errors through cross-verification of results while compensating for *matK* amplification failures in certain taxa [23] and *rbcL*‘s limited discriminatory capacity [27]. Given their complementary benefits, the Consortium for the Barcode of Life (CBOL) recommends combining both genes as the standard land plant barcode [25], offering optimal universality, sequence quality, and cost-effectiveness. This dual-gene approach has achieved successful species identification in 72% of cases.

At the hive level, Hive 2 displayed the highest diversity of taxa, likely reflecting greater heterogeneity in the pollen sources collected by bees. Concerning the variation in genera across hives, this may be due to microenvironmental differences or variations in the bees’ foraging behavior [4], considering that *A. mellifera* is a species characterized by being polylectic [28], which could be related to the richness of the pollen material found. The most frequent genera in the bee bread samples, belonging to the Cactaceae family, may be related to their notable diversification in arid and semi-arid environments, as suggested by Hernández-Hernández et al. [29], who highlight that this high diversity results from various ecological and evolutionary factors. Additionally, this cactus family exhibits attractive flowering strategies for a wide range of pollinator groups, including bees [30], as shown in a recent study on the genus *Opuntia*, where *A. mellifera* was one of the most effective pollinators [31].

On the other hand, the detection of certain plant families with distributions restricted to other regions could be attributed to the limitations of non-local reference databases, in which many taxa lack available molecular information [32,33]. Likewise, potential matches with closely related taxa and the scarcity of comprehensive studies on local flora should be considered, as these factors may also contribute to the observed results.

Overall, the results demonstrated the potential of the DNA barcoding approach to recover a wide range of plant genera compared to the microscopy technique. These partial discrepancies between the techniques were likely due to the mixture of pollen samples and the sensitivity of the two methods. While microscopy enables the direct visualization of pollen morphology, molecular approaches can detect traces of plant DNA from pollen, including taxa that are underrepresented or morphologically indistinguishable under the microscope, which could be resolved with expert personnel [9,21]. This was one of the main challenges of this kind of analysis. Therefore, combining both methods provided a more comprehensive picture of the botanical composition of bee bread.

This limitation highlighted the need to implement alternative methods to disentangle the floral composition of honey bee pollen, such as TA cloning of PCR products, which enables cloning of amplicons using vectors for subsequent individual sequencing [34]. Another approach for mixed samples involves high-throughput sequencing techniques like metabarcoding, which allows identification of multiple taxa [16,35]—not only plants but also other organisms, including fungi and bacteria [36]. However, Sanger sequencing offered a cost-effective and straightforward approach that can be used as a preliminary tool to support the development of local honey brands by validating that their products originate primarily from the biodiversity of the tropical dry forest, thereby adding both ecological and market value [37]. In Colombia, sequencing six samples using the Sanger method costs about 225 USD. In contrast, processing the same number of samples through metabarcoding costs around 2550 USD. Therefore, Sanger sequencing is considerably more cost-effective, reducing expenses by nearly 91%. Therefore, for local farmers, Sanger sequencing could be informative and could add market value to the product. In this sense, the implementation of fast and cost-effective methodologies represents an opportunity for local beekeepers and farmers. These tools allow them not only to add value by authenticating their apicultural products but also to contribute to their food security and sovereignty. This is achieved by guaranteeing traceability, quality, and commercial value, thereby strengthening their productive autonomy while simultaneously enabling the utilization of local biodiversity. Although the beekeeping sector has been increasing in the last decade, the legislation still shows significant gaps. Recent studies in the Colombian Caribbean [38] emphasized the need for clearer regulations to support value-added honey products, as well as botanical and geographical certifications, as have been used in other countries [37].

## 4. Materials and Methods

### 4.1. Study Area

The study was conducted at La Paz Verso solar mini-farm (Solenium, La Paz, Cesar, Colombia), a sustainable solar energy production facility located at coordinates 10.4° N, −73.2° W (Los Robles rural district, La Paz municipality, Cesar Department, Colombia). The 9.6-ha site exhibits a vegetation cover composed of pasture-crop mosaics with dominant stands of *Prosopis juliflora* (*i.e.*, *trupillo*) [39]. This dry forest area, classified under Holdridge’s life zone system [40], contains an apiary of eight (8) beehives associated with a conservation project. The ecosystem is characterized by mean annual temperatures of 24 °C–26 °C and bimodal-tetraseasonal rainfall (894 mm/year–1800 mm/year), featuring two rainy seasons (April-June and August-November) separated by a mid-year dry spell (*veranillo*) in July and an extended dry period from December to March [41].

### 4.2. Pollen Collection

**Figure 6:**
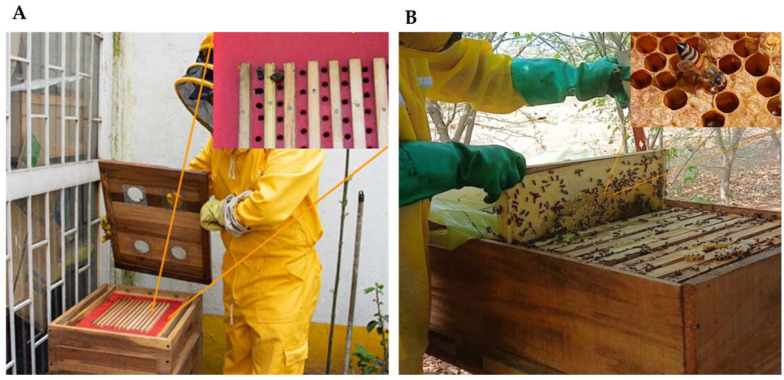
Commonly used beehives in apiculture. (**A**) A hive with upper pollen traps, which are the most commonly used traps to collect pollen. Figure adapted from [46] with minor modifications. (**B**) Langstroth-type hive composed of 35 movable frames, from which the bee bread samples were extracted in this study.

Samples were collected in April by cutting comb fragments containing bee bread from three *A. mellifera* hives randomly selected (see Figure 6B). In the laboratory, bee bread was extracted from the comb cells of each sampled fragment. A commercial dehydrated pollen sample was also included to compare DNA yield and quality with that extracted from bee bread. This pollen was obtained using pollen traps installed at the hive entrance, which induce the removal of pollen loads from returning foragers [42], allowing collection of pollen samples for research or commercial use without significantly affecting the colony’s food supply (see Figure 6A). The collected pollen was then dehydrated at temperatures ranging from 32 °C [43] to 42 °C [44]—a critical factor influencing the preservation of its chemical composition [45]. All samples were preserved undivided in 1.5 mL tubes at −80 °C to prevent compositional changes.

### 4.3. Evaluation of Pollen Grain Rupture Methods

Replicated assays were performed with the samples to standardize DNA extraction conditions from pollen, aiming to obtain high-quality DNA suitable for subsequent analyses. To ensure disruption of the pollen grains’ resistant outer layer (exine) [47], combined DNA extraction techniques were required [21]. Three mechanical methods were evaluated to facilitate pollen grain rupture, based on comparisons of yield, integrity, and purity. The methods consisted of: (1) agitation with glass beads (3 mm–5 mm), (2) cordless homogenizer for microtubes (Bel-Art™ ProCulture™, USA), and (3) maceration with a sterilized porcelain mortar. These methods were selected because they have been evaluated in previous studies, demonstrating effectiveness for cellular disruption and DNA release [8,21,22,48]. Prior to processing, samples were frozen at −80 °C for 24 h.

### 4.4. DNA Extraction

In total, three independent samples, each corresponding to a different beehive, were analyzed. DNA was extracted from each sample and processed in duplicate as technical replicates. The BioSpin Plant Genomic DNA Extraction Kit (BSC13S1, BioFlux, Belgium) was used, following the manufacturer’s instructions. Briefly, 150 mg of pollen samples were homogenized with 450 μL of lysis buffer provided in the kit. The mixture was incubated at 65 °C for 15 min, and then 150 μL of DA buffer was added. After centrifugation (at 12,000× *g* for 5 min) in a Thermo Scientific Microcentrifuge (model MICRO CL21R, Germany), the supernatant was transferred to a new tube, where 1.5 volumes of P binding buffer were added. Subsequently, 750 μL of the mixture was transferred to the filter column (Spin Column), centrifuged (at 12,000× *g* for 1 min), and the flow-through was discarded. A wash was performed with 500 μL of G binding buffer solution, followed by two washes with 600 μL of washing buffer. Elution was carried out in 20 μL of elution buffer.

### 4.5. PCR and Sequencing

PCR amplification was performed on DNA extracted from bee hives. Two molecular markers were used, the maturase K (*matK*) and ribulose-1,5-bisphosphate carboxylase (*rbcL*) genes (see Supplementary Materials, Table S3) for the molecular plant taxon identification. These functional chloroplast genes were recommended by CBOL as the barcode for land plants [25]. The amplification of the target genes was carried out in a T100 Thermal Cycler (BIORAD, USA). The PCR reaction mixture was prepared with a final volume of 20 μL, containing: RNase-free H_2_O, 1 × Taq buffer, dNTP mix at 0.25 mM, forward and reverse primers each at 0.5 μM, ExcelTaq^TM^ (TP1000, SMOBIO, Taiwan), and the DNA of interest. Negative controls with RNase-free H_2_O were included. The reagents were used in the amounts indicated by the manufacturer for the enzyme used.

The thermal cycling conditions for the *rbcL* region were as follows: initial denaturation at 94 °C for 3 min; 35 cycles of denaturation at 94 °C for 30 s, annealing at 53 °C for 40 s, and extension at 72 °C for 50 s; followed by a final extension at 72 °C for 5 min. In the case of *matK*, the protocol was optimized by adjusting the annealing temperatures, as amplification under the same conditions used for *rbcL* was unsuccessful. Therefore, two gradient PCRs (50 °C to 60 °C and 45 °C to 60 °C) were performed to determine the optimal annealing temperature, based on the approach evaluated by Yu et al. [23].

PCR products were confirmed on a 1% agarose gel in 1× TAE buffer, using FluoroVue™ Nucleic Acid Gel Stain (10,000×) as an intercalating agent, following the manufacturer’s instructions. Electrophoresis was performed at 80 V for 45 min. The expected fragments—approximately 580 bp [49] to 800 bp [50] for *rbcL* and 772 bp [23] to 850 bp in length for *matK* [49]—were verified using an ultraviolet light transilluminator (model GEL DOC GO, BIORAD, Singapore). The samples were subsequently sent for purification and sequencing using Sanger sequencing methods.

### 4.6. Microscopic Analysis of Pollen

For the morphological observation of pollen grains, two initial washes with cold distilled water were performed. The washes were added to 1.5 mL microcentrifuge tubes containing bee bread samples (pollen source), centrifuged at 1000× *g* for 3 min, and the supernatant was discarded. A third wash was carried out with 50% cold ethanol, followed by centrifugation (1000× *g* for 3 min) and removal of the supernatant. For slide preparation, a small amount of the sample was placed on a glass slide, and a drop of 50% glycerin–water solution was added and left for 3 min. Subsequently, a drop of 0.05% fuchsin in 50% glycerin–water was added and left for 5 min. Observations were then made using an Olympus EP50 inverted microscope (Tokyo, Japan) at a total magnification of 400×.

To approximate the taxonomic identity of the pollen types, reference materials were consulted, including the Red de Catálogos Polínicos online (RCPol), PalDat—Palynological Database, da Silva et al. [51], García et al. [52], and Montoya-Pfeiffer et al. [53].

### 4.7. Data Analysis

Following sequencing, chromatograms were individually edited using CHROMAS 2.6.6 software to obtain high-quality sequences. Sequence evaluation (forward and reverse) involved trimming low-quality ends based on observed peak patterns. This process was performed independently for each amplified gene (*rbcL* and *matK*). The resulting sequences were compared against the reference database using the Basic Local Alignment Search Tool (BLAST+ 2.17.0) from the National Center for Biotechnology Information (NCBI) to determine sequence identities. To ensure higher reliability in taxonomic assignment at the genus level, only sequences with ≥80% identification match were considered.

From the identified genera, the number of taxa per hive was estimated, along with shared and unique taxa. Additionally, taxon recovery efficiency was evaluated for each gene (*matK* and *rbcL*) by comparing the number and type of genera detected. Likewise, the most predominant families were highlighted based on the genera with the highest occurrence.

## 5. Conclusions

The importance of adjusting protocols according to the intrinsic characteristics of pollen samples and their origin is highlighted, with the aim of establishing efficient methodologies for the molecular identification of plant species. There is great interest in honey-derived products in agriculture due to their reported health benefits, which depend largely on the composition of the pollen collected by bees [54]. To validate the botanical origin of these species and confirm the floral sources from which honey is produced, barcoding studies are required.

To achieve this objective, the results of this study confirmed the practicality of this approach. In general, bee bread proved to be a viable source of plant DNA, with yield variations that may be associated with the very nature of the mixed sample. Despite this, *matK* and *rbcL* proved effective for genus-level identification through DNA barcoding, albeit with differences in performance. Although this study was based on a limited number of biological replicates (three hives), which restricts statistical inference, it provides valuable preliminary and descriptive evidence supporting the feasibility of a cost-effective molecular approach to identify the botanical origin of mixed plant DNA in bee bread. These exploratory results suggest that combining *rbcL* and *matK* markers can enhance taxonomic resolution and serve as a foundation for future, more comprehensive analyses. Also, future studies should implement other techniques such as TA cloning or metabarcoding, which allow for a better approach to detecting multiple taxa at the species level. Furthermore, these results could support the development of a molecular reference database for the floral composition of *A. mellifera* hives in tropical dry forests, enhancing future studies on plant–pollinator interactions and honey authentication for local honey producers.

## Figures and Tables

**Table 1:** Quantification of DNA extracted from pollen trap samples.

Method	DNA (ng/μL)	A260/A280	A260/A230
Mortar	1157	2.09	1.97
Glass beads	380	2.08	2.07
Cordless Homogenizer	1241	2.09	2.04

**Table 2:** Comparison of DNA yield and purity indices extracted from different types of pollen source samples.

Sample Origin	Samples Type	DNA (ng/μL)	A260/A280	A260/A230
Hive 1	Bee bread	1387 ± 341	2.14 ± 0.01	2.21 ± 0.05
Hive 2	Bee bread	910 ± 148	2.12 ± 0.01	2.22 ± 0.03
Hive 3	Bee bread	1142 ± 289	2.12 ± 0.01	2.15 ± 0.05
Commercial pollen	Dry pollen	327 ± 72	1.96 ± 0.01	1.89 ± 0.04

**Table 3:** Comparative BLAST statistics from the top 20 hits for *matK* and *rbcL* gene regions from mixed plant DNA sequences derived from bee bread.

Sample Origin	*matK*			*rbcL*		
	Percent Sequence Identity (%)	E-Value	Total Score	Percent Sequence Identity (%)	E-Value	Total Score
Hive 1	97.0 ± 1.1	0.0 ± 0.0	1235 ± 36	98.0 ± 0.9	0.0 ± 0.0	1437 ± 36
Hive 2	89.0 ± 8.0	2.3 × 10^−114^ ± 2.0 × 10^−114^	826 ± 382	99.0 ± 0.6	0.0 ± 0.0	1489 ± 8
Hive 3	97.0 ±0.5	0.0 ± 0.0	1208 ± 28	99.0 ± 0.1	0.0 ± 0.0	1480 ± 11

**Table 4:** Main BLAST matches with their identity values and GenBank accession numbers.

Top Hit Genus	Percent Query Coverage (%)	Percent Sequence Identity (%)	Total Score	Assigned Accession Number
*Leuenbergeria*	99	99	1373	AY875242.1
*Rhodocactus*	99	99	1359	AY875244.1
*Pereskia*	98	98	1313	OR711232.1

## Data Availability

The raw data supporting the conclusions of this article will be made available by the authors on request.

## Supplementary Materials

The following supporting information can be downloaded at https://www.mdpi.com/article/10.3390/plants14233652/s1. Table S1: Genus-level taxa identified from bee bread samples using DNA barcoding with the *matK* and *rbcL* markers; Table S2: Plant taxa at the genus level identified per beehive and shared among them; Table S3: Primer information. Figures S1 and S2: Stained palynomorphs with fuchsin, obtained from a bee bread sample collected from the hives. The possible taxonomic level identified at the family level is indicated.
