# Supplementary material for: Uncovering the Floral Origins of Honey Bee Pollen in Colombian Tropical Dry Forest: A Low-Cost DNA Barcoding Approach Reveals Cactaceae Dominance
Source: Plants (Basel). 2025 Nov 30;14(23):3652. doi: 10.3390/plants14233652 (PMC12694352; doi:10.3390/plants14233652)
Supplement: Supplementary file 1 [file plants-14-03652-s001.zip › plants-3975154-supplementary.pdf]

plants

MDPI

Table S1. Genus level taxa identified from bee bread samples using DNA barcoding with the *matK* and *rbcL* markers. Families marked with * correspond to those restricted to other regions (outside Colombia).

Family	Genus	*matK*		*rbcL*		Category Marker
		Abundance	%	Abundance	%	
Cactaceae	*Airampoa*	0	0.0	12	3.3	Unique to *rbcL*
Didiereaceae*	*Alluaudia*	0	0.0	2	0.5	Unique to *rbcL*
Ancistrocladaceae*	*Ancistrocladus*	1	0.4	0	0.0	Unique to *matK*
Amaranthaceae	*Archiatriplex*	1	0.4	0	0.0	Unique to *matK*
Cactaceae	*Ariocarpus*	8	3.4	0	0.0	Unique to *matK*
Cactaceae	*Astrophytum*	3	1.3	0	0.0	Unique to *matK*
Amaranthaceae	*Atriplex*	1	0.4	0	0.0	Unique to *matK*
Cactaceae	*Austrocylindropuntia*	8	3.4	12	3.3	Shared (*matk & rbcL*)
Cactaceae	*Aztekium*	2	0.8	0	0.0	Unique to *matK*
Barbeuiaceae*	*Barbeuia*	0	0.0	5	1.4	Unique to *rbcL*
Basellaceae	*Basella*	0	0.0	1	0.3	Unique to *rbcL*
Cactaceae	*Brachycereus*	3	1.3	0	0.0	Unique to *matK*
Cactaceae	*Brasiliopuntia*	0	0.0	12	3.3	Unique to *rbcL*
Cactaceae	*Browningia*	0	0.0	3	0.8	Unique to *rbcL*
Montiaceae	*Calandrinia*	1	0.4	0	0.0	Unique to *matK*
Cactaceae	*Calymmanthium*	1	0.4	12	3.3	Shared (*matk & rbcL*)
Cactaceae	*Cereus*	0	0.0	3	0.8	Unique to *rbcL*
Amaranthaceae	*Chenopodiastrum*	1	0.4	0	0.0	Unique to *matK*
Amaranthaceae	*Chenopodium*	1	0.4	0	0.0	Unique to *matK*
Montiaceae	*Cistanthe*	1	0.4	1	0.3	Shared (*matk & rbcL*)
Fabaceae	*Conzattia*	1	0.4	0	0.0	Unique to *matK*
Cactaceae	*Copiapoa*	0	0.0	11	3.0	Unique to *rbcL*
Lophiocarpaceae	*Corbichonia*	0	0.0	1	0.3	Unique to *rbcL*
Cactaceae	*Cumulopuntia*	8	3.4	0	0.0	Unique to *matK*
Cactaceae	*Deamia*	1	0.4	0	0.0	Unique to *matK*
Didiereaceae*	*Didierea*	0	0.0	1	0.3	Unique to *rbcL*
Cactaceae	*Echinocactus*	9	3.8	11	3.0	Shared (*matk & rbcL*)
Cactaceae	*Echinocereus*	8	3.4	0	0.0	Unique to *matK*
Cactaceae	*Epiphyllum*	8	3.4	0	0.0	Unique to *matK*
Cactaceae	*Epithelantha*	1	0.4	0	0.0	Unique to *matK*
Cactaceae	*Eriosyce*	4	1.7	11	3.0	Shared (*matk & rbcL*)
Amaranthaceae	*Exomis*	1	0.4	0	0.0	Unique to *matK*
Amaranthaceae	*Extriplex*	1	0.4	0	0.0	Unique to *matK*
Cactaceae	*Ferocactus*	9	3.8	11	3.0	Shared (*matk & rbcL*)
Anacampserotaceae	*Grahamia*	0	0.0	1	0.3	Unique to *rbcL*
Malvaceae	*Grewia*	1	0.4	0	0.0	Unique to *matK*
Nyctaginaceae	*Guapira*	0	0.0	10	2.7	Unique to *rbcL*

Asteraceae	*Gynura*	0	0.0	1	0.3	Unique to *rbcL*
Dioncophyllaceae*	*Habropetalum*	1	0.4	0	0.0	Unique to *matK*
Halophytaceae*	*Halophytum*	0	0.0	1	0.3	Unique to *rbcL*
Cactaceae	*Hatiora*	8	3.4	12	3.3	Shared (*matk & rbcL*)
Fabaceae	*Heteroflorum*	1	0.4	0	0.0	Unique to *matK*
Cactaceae	*Homalocephala*	9	3.8	0	0.0	Unique to *matK*
Cactaceae	*Jasminocereus*	4	1.7	0	0.0	Unique to *matK*
Cactaceae	*Kadenicarpus*	8	3.4	0	0.0	Unique to *matK*
Cactaceae	*Lepismium*	0	0.0	12	3.3	Unique to *rbcL*
Cactaceae	*Leuchtenbergia*	7	3.0	9	2.5	Shared (*matk & rbcL*)
Cactaceae	*Leuenbergeria*	8	3.4	12	3.3	Shared (*matk & rbcL*)
Lophiocarpaceae	*Lophiocarpus*	0	0.0	1	0.3	Unique to *rbcL*
Montiaceae	*Lyallia*	1	0.4	0	0.0	Unique to *matK*
Cactaceae	*Lymanbensonia*	8	3.4	0	0.0	Unique to *matK*
Cactaceae	*Maihuenia*	1	0.4	12	3.3	Shared (*matk & rbcL*)
Amaranthaceae	*Manochlamys*	1	0.4	0	0.0	Unique to *matK*
Cactaceae	*Melocactus*	0	0.0	8	2.2	Unique to *rbcL*
Malvaceae	*Microcos*	1	0.4	0	0.0	Unique to *matK*
Amaranthaceae	*Microgynoecium*	1	0.4	0	0.0	Unique to *matK*
Cactaceae	*Miqueliopuntia*	0	0.0	12	3.3	Unique to *rbcL*
Cactaceae	*Neoraimondia*	2	0.8	0	0.0	Unique to *matK*
Cactaceae	*Neowerdermannia*	8	3.4	0	0.0	Unique to *matK*
Cactaceae	*Nopalea*	0	0.0	12	3.3	Unique to *rbcL*
Cactaceae	*Opuntia*	1	0.4	12	3.3	Shared (*matk & rbcL*)
Cactaceae	*Parodia*	4	1.7	11	3.0	Shared (*matk & rbcL*)
Cactaceae	*Pereskia*	9	3.8	12	3.3	Shared (*matk & rbcL*)
Cactaceae	*Pereskiopsis*	1	0.4	7	1.9	Shared (*matk & rbcL*)
Cactaceae	*Pfeiffera*	1	0.4	0	0.0	Unique to *matK*
Montiaceae	*Phemeranthus*	1	0.4	1	0.3	Shared (*matk & rbcL*)
Didiereaceae*	*Portulacaria*	0	0.0	12	3.3	Unique to *rbcL*
Cactaceae	*Pterocactus*	1	0.4	0	0.0	Unique to *matK*
Cactaceae	*Quiabentia*	1	0.4	0	0.0	Unique to *matK*
Cactaceae	*Rhipsalis*	8	3.4	12	3.3	Shared (*matk & rbcL*)
Cactaceae	*Rhodocactus*	8	3.4	12	3.3	Shared (*matk & rbcL*)
Cactaceae	*Rimacactus*	0	0.0	12	3.3	Unique to *rbcL*
Cactaceae	*Salmonopuntia*	0	0.0	12	3.3	Unique to *rbcL*
Cactaceae	*Schlumbergera*	0	0.0	12	3.3	Unique to *rbcL*
Cactaceae	*Sclerocactus*	8	3.4	0	0.0	Unique to *matK*
Cactaceae	*Selenicereus*	1	0.4	0	0.0	Unique to *matK*
Caryophyllaceae	*Silene*	1	0.4	0	0.0	Unique to *matK*
Stegnosperma-taceae*	*Stegnosperma*	0	0.0	2	0.5	Unique to *rbcL*
Cactaceae	*Stenocactus*	3	1.3	0	0.0	Unique to *matK*
Cactaceae	*Stenocereus*	4	1.7	0	0.0	Unique to *matK*
Cactaceae	*Strombocactus*	8	3.4	0	0.0	Unique to *matK*
Amaranthaceae	*Stutzia*	1	0.4	0	0.0	Unique to *matK*
Cactaceae	*Tacinga*	1	0.4	11	3.0	Shared (*matk & rbcL*)

Talinaceae	*Talinella*	0	0.0	7	1.9	Unique to *rbcL*
Talinaceae	*Talinum*	0	0.0	12	3.3	Unique to *rbcL*
Fabaceae	*Templetonia*	1	0.4	0	0.0	Unique to *matK*
Cactaceae	*Tephrocactus*	3	1.3	0	0.0	Unique to *matK*
Cactaceae	*Thelocactus*	9	3.8	0	0.0	Unique to *matK*
Dioncophyllaceae*	*Triphyophyllum*	1	0.4	0	0.0	Unique to *matK*
Cactaceae	*Turbinicarpus*	8	3.4	0	0.0	Unique to *matK*
Cactaceae	*Weingartia*	0	0.0	5	1.4	Unique to *rbcL*
Cactaceae	*Xiquexique*	0	0.0	3	0.8	Unique to *rbcL*
Total		**237**	**100**	**367**	**100**	-

Table S2. Plant taxa at the genus level identified per beehive and shared among them.

Category	Genus
Shared genus between the three beehives	*Leuenbergeria, Rhodocactus, Pereskia, Strombocactus, Homalocephala, Thelocactus, Ariocarpus, Neowerdermannia, Turbinicarpus, Kadenicarpus, Sclerocactus, Echinocactus, Austrocylindropuntia, Rhipsalis, Echinocereus, Lymanbensonia,Maihuenia, Ferocactus, Epiphyllum, Cumulopuntia, Hatiora, Leuchtenbergia, Opuntia, Tacinga, Astrophytum, Stenocactus, Portulacaria, Talinum, Calymmanthium, Talinella, Schlumbergera, Lepismium, Rimacactus, Copiapoa, Parodia, Eriosyce, Miqueliopuntia, Pereskiopsis, Salmonopuntia, Guapira, Melocactus, Airampoa, Brasiliopuntia, Nopalea, Barbeuia, Weingartia*
Beehive 1 only	*Basella, Corbichonia, Didierea, Grahamia, Gynura, Halophytum, Lophiocarpus, Pterocactus, Stegnosperma*
Beehive 2 only	*Ancistrocladus, Archiatriplex, Atriplex, Calandrinia, Chenopodiastrum,, Chenopodium, Conzattia, Epithelantha, Exomis, Extriplex, Grewia,, Habropetalum, Heteroflorum, Lyallia, Manochlamys, Microcos, Microgynoecium, Pfeiffera, Quiabentia, Selenicereus, Silene, Stutzia, Templetonia, Triphyophyllum*
Beehive 3 only	*Deamia*
Beehive 1 and 2	*Alluaudia, Aztekium, Browningia, Cistanthe, Phemeranthus*
Beehive 1 and 3	*Brachycereus, Jasminocereus, Tephrocactus*
Beehive 2 and 3	*Cereus, Neoraimondia, Stenocereus, Xiquexique*

Table S3. Primers information.

Gene	Primer	Sequence 5' - 3'	Melting temperature (°C)	Product size (bp)	Reference
Ribulose-1,5-bisphosphate carboxylase (*rbcL*)	rbcL_F	AGACCTWTTTGAAGAAGGTTCWGT	59.6	700	[50]
	rbcL_R	TCGGTYAGAGCRGGCATRTGCCA	66.0		
Maturase kinase (matK)	matK_F	CCCRTYCATCTGGAAATCTTGGTTC	66.0	720	[23]
	matK_R	GCTRTRATAATGAGAAAGATTCTGC	54.2		

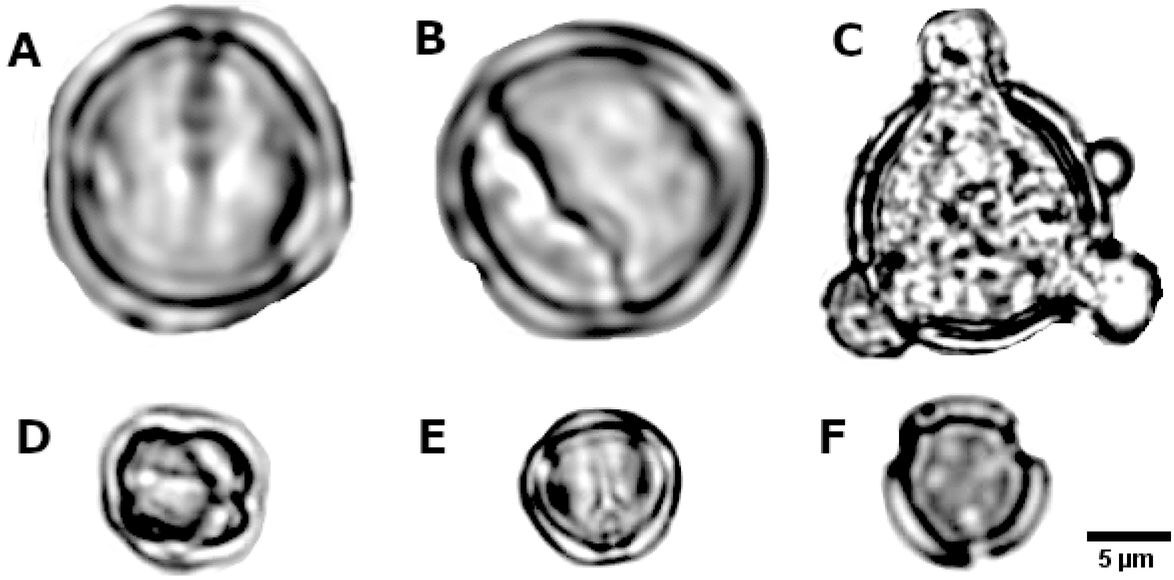

Figure S1. Stained palynomorphs with fuchsin, obtained from a bee bread sample collected from the hives. The possible taxonomic level identified at the family level is indicated. (**A**)**.** Vitaceae. (**B**)**.** Cucurbitaceas**.** (**C**). Asteraceas / Malvaceae. (**D**). Asteraceas / Amaranthaceae .(**E**). Asteraceas. (**F**). Fabaceae**.** Total magnification: 400×.

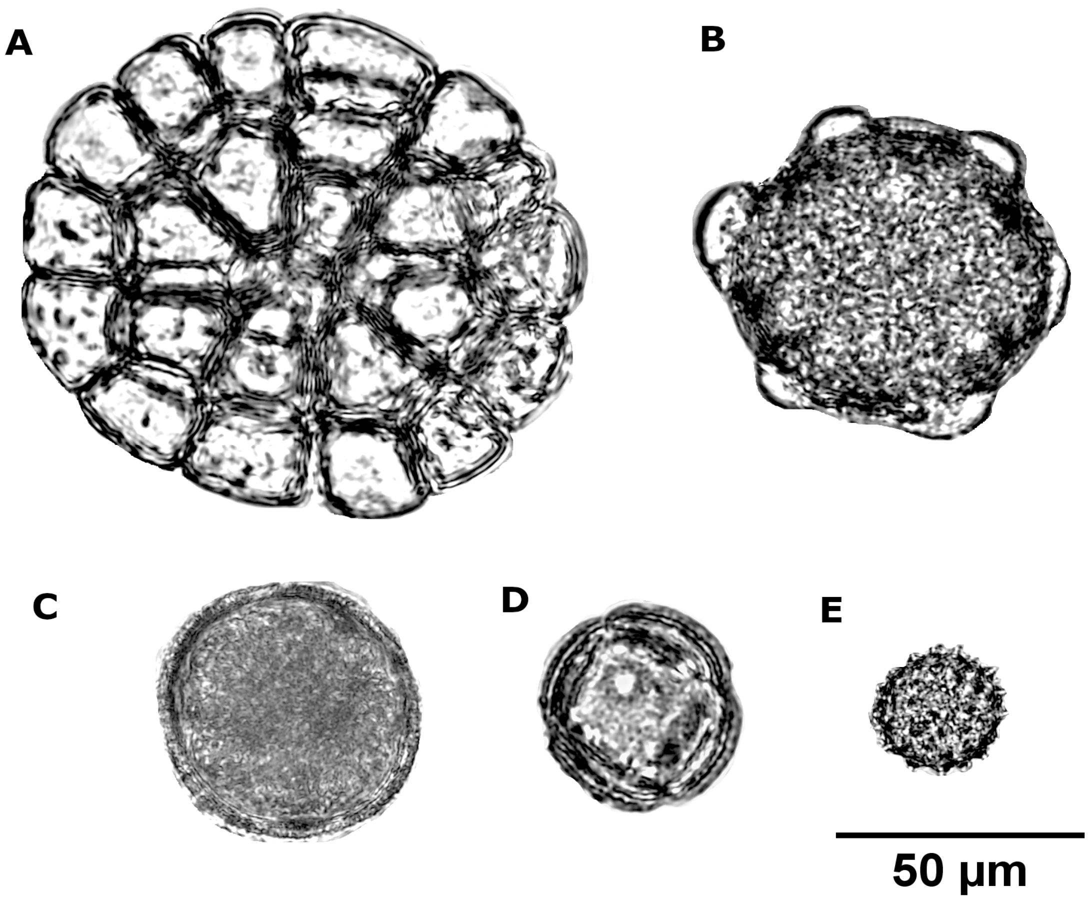

Figure S2. Stained palynomorphs with fuchsin, obtained from a bee bread sample collected from the hives. The possible taxonomic level identified at the family level is indicated. (**A**). Fabaceae (Mimosoidae). (**B**)**.** Cactaceae (**C**)**.** Asteráceas / Nyctaginaceae (**D**)**.** Lamiaceae. (**E**)**.** Asteraceae / Malvaceae. Total magnification: 400×.
